# A Scene Detection Complexity Metric for Infrared Small Target Detection

**DOI:** 10.3390/s26092886

**Published:** 2026-05-05

**Authors:** Zhiyuan Huang, Zhiyong Zhang

**Affiliations:** School of Electronics and Communication Engineering, Sun Yat-sen University, Shenzhen 518107, China; huangzhy73@mail2.sysu.edu.cn

**Keywords:** infrared small target detection, scene detection complexity, objective weighting, entropy weight method, principal component analysis, performance evaluation

## Abstract

Infrared small target detection is widely used in aerospace surveillance, maritime search and rescue, and military reconnaissance. However, the performance of detection algorithms is highly dependent on scene characteristics, and methods that perform well in simple backgrounds may degrade substantially in complex environments. Existing indicators, such as information entropy, average gradient, and peak signal-to-noise ratio, can reflect detection difficulty from individual perspectives, but they do not provide a unified measure that jointly considers target saliency, background complexity, and target–background coupling. To address this issue, this study proposes a scene detection complexity (SDC) metric for quantifying the difficulty of infrared small target detection. Six basic indicators are selected from three dimensions, namely target saliency, background complexity, and target–background coupling: statistical variance, target–background contrast, signal-to-clutter ratio, information entropy, structural similarity, and target size. After Min–Max normalization, objective weights are determined by combining the entropy weight method and principal component analysis, and the weighted indicators are fused into an SDC value in the range of [0,1]. Experiments on 100 test images selected from IRST640, MSISTD, SIRST-V2, and an infrared small-aircraft sequence dataset show that the proposed SDC achieves a Pearson linear correlation coefficient of 0.956 with subjective difficulty ratings and −0.902 with image-level detection scores obtained from seven representative algorithms. The results further indicate that traditional methods are more sensitive to increasing scene complexity, whereas deep-learning-based methods are comparatively more robust in complex backgrounds. The proposed SDC provides a unified and objective tool for performance evaluation, algorithm selection, and pre-assessment of scene difficulty in infrared small target detection.

## 1. Introduction

Infrared small target detection has attracted considerable attention in recent years because of its importance in aerospace observation, remote sensing, maritime rescue, and security monitoring [1,2]. Compared with visible imaging, infrared sensing offers unique advantages, including all-day operation, insensitivity to illumination variations, and long-range perception capability. These characteristics make it an important technical route for target detection in complex environments.

Despite these advantages, infrared small target detection remains challenging. Small targets usually occupy only a few pixels, contain little texture or shape information, and often appear with low contrast and weak signals. Under strong clutter backgrounds, such as sea waves, dense clouds, or urban structures, the statistical and structural similarity between the target and the background increases substantially, which makes the target more likely to be submerged by interference [3]. As a result, the same algorithm may exhibit markedly different performance across different scenes.

With the rapid development of infrared small target detection algorithms, objective and comprehensive performance evaluation has become increasingly important for both algorithm optimization and engineering deployment [4,5]. Existing evaluation approaches are usually divided into qualitative and quantitative categories. Qualitative evaluation relies on visual inspection of detection results, whereas quantitative evaluation commonly uses false alarm rate, miss rate, detection probability, and area under the curve. However, these metrics mainly describe how an algorithm performs on a given scene, rather than how difficult the scene itself is for detection. Consequently, conclusions drawn from a small number of test images may not fully reveal the intrinsic performance boundary or generalization capability of a detection method.

Research on image quality assessment provides a useful perspective for this problem. Depending on whether human visual judgment is involved, image quality assessment can be categorized into subjective and objective methods [6,7]. Subjective methods, such as mean opinion score, generally align well with human perception but are time-consuming and costly. Objective methods estimate quality through mathematical models and are therefore more suitable for large-scale and repeatable evaluation. This line of research suggests that the difficulty of a visual task can be quantified through scene-related image characteristics rather than being inferred only indirectly from final algorithm outputs.

Recent studies have attempted to connect infrared detection difficulty with scene characteristics. Zheng et al. introduced global background dissimilarity and local background saliency to characterize the detectability of sea-surface infrared targets [8]. Sun proposed gradient information entropy to describe background texture complexity [9]. Quan et al. fused multiple features using a random forest model to evaluate the quality of simulated infrared images [10]. Deng Baoyuan et al. [11] proposed a multi-modal fusion target recognition method called YCNNet, which is used to address the issue of insufficient sensing capability of a single sensor in complex road scenes at night. Although these studies provide valuable insights, most existing approaches focus on a single feature or a specific aspect of the problem. A unified metric that jointly considers target saliency, background complexity, and target–background coupling is still lacking.

To address this gap, this paper proposes a scene detection complexity (SDC) metric for infrared small target detection. The proposed metric establishes a six-dimensional feature description system composed of statistical variance (SV), target–background contrast (TBC), signal-to-clutter ratio (SCR), information entropy (*H*), structural similarity (SSIM), and target size (*S*). After Min–Max normalization, the entropy weight method and principal component analysis (PCA) are combined to determine the final indicator weights, and the weighted features are fused into a scalar complexity index with values in [0,1]. The proposed SDC fundamentally differs from conventional single-indicator descriptors in both representation scope and evaluation objective. Traditional indicators, such as entropy, contrast, average gradient, and SCR, usually quantify only one local property of the image or one specific aspect of target detectability. However, the actual difficulty of infrared small target detection is governed by multiple coupled factors, including the prominence of the target itself, the clutter level of the background, and the similarity between target and background structures. As a result, no single indicator can consistently describe scene difficulty across diverse infrared scenarios. The proposed SDC addresses this limitation by constructing a unified multi-indicator framework and combining entropy-based weighting with PCA-based weighting to account for both indicator discriminability and inter-feature correlation. In this sense, the contribution of SDC is not simply to aggregate existing features, but to provide a scene-centered and quantitatively interpretable metric for assessing infrared detection difficulty in a more general and robust manner.

The significance of such a metric is threefold. First, it helps delineate the performance boundary of different algorithms under different scene conditions. Second, it provides a more objective basis for relating scene characteristics to detection performance, thereby supporting algorithm selection. Third, it enables rapid pre-assessment of detection difficulty before algorithm deployment, which can reduce trial-and-error costs in practical systems.

The main contributions of this work are summarized as follows:

1. A multidimensional description framework for infrared small target detection difficulty is established from the perspectives of target saliency, background complexity, and target–background coupling, using six basic indicators: SV, TBC, SCR, *H*, SSIM, and *S*.
2. An objective weighting strategy that combines the entropy weight method and PCA is introduced to balance indicator discrimination and correlation structure, yielding more robust composite weights.
3. Experiments are conducted on 100 test images selected from IRST640, MSISTD, SIRST-V2, and an infrared small-aircraft sequence dataset. All correlation analyses in this paper are performed on the full set of 100 images rather than on illustrative examples only.
4. The proposed SDC is used to analyze performance variation of traditional and deep-learning-based methods across scenes of different complexity, providing quantitative support for algorithm evaluation and selection.

## 2. Related Work

### 2.1. Infrared Small Target Detection Methods

Infrared small target detection has been extensively studied in recent years. A review by Rawat et al. summarized the recent development of infrared small target detection algorithms and highlighted the long-standing importance of contrast enhancement and clutter suppression in complex backgrounds [12]. Zhao et al. further provided a comprehensive survey of single-frame infrared small-target detection and showed that the field has gradually evolved from handcrafted local-feature methods to model-driven and deep-learning-based approaches [13]. More recently, deep neural network models such as RISTDnet have demonstrated the potential of data-driven representations for improving robustness in challenging infrared scenes [14].

Despite these advances, most existing studies mainly focus on improving detector performance itself, rather than establishing a unified metric to quantify the intrinsic difficulty of different infrared scenes. This limitation motivates the development of a scene-level complexity measure for infrared small target detection.

### 2.2. Scene Difficulty and Complexity Indicators

Understanding and quantifying scene difficulty has been a long-standing concern in the infrared target detection community. Early efforts primarily relied on single, handcrafted descriptors such as information entropy, average gradient, and signal-to-clutter ratio (SCR) to characterize specific aspects of an image, like background texture or target contrast. While computationally efficient, these metrics capture only isolated properties and often fail to correlate robustly with actual detection performance across diverse scenarios.

Recognizing the limitations of single metrics, several studies have moved toward multi-dimensional evaluation frameworks. For instance, Zheng et al. proposed a criterion based on scene features, introducing the Interference Degree of Global Background (IDGB) and the Similarity Degree of Local Background (SDLB) to jointly assess image quality for target detection [15]. Similarly, in the context of Automatic Target Recognition (ATR), researchers like Wang et al. have defined complexity from the perspectives of target-clutter similarity and submergence degree, integrating multiple features through weighted processing [16]. These works represent a critical shift toward a more holistic and task-oriented evaluation, moving beyond simple image statistics.

Parallel to these efforts, a significant body of work has focused specifically on quantifying background clutter, a primary factor in detection difficulty. A recent comprehensive review by VonNiederhausern (2023) underscores a key challenge in the field: “there is not a standardized metric for quantifying background clutter” and many existing metrics have not been validated for point-source targets like those in infrared small target detection [17]. This finding highlights a persistent gap that a unified complexity metric could help address.

More recently, the scope of complexity assessment has begun to evolve from static, single-frame analysis toward dynamic, sequence-level evaluation. The work by Wang et al. is a notable example, proposing a method to measure the complexity of an entire infrared image sequence for automatic target tracking. Their research is grounded in the critical observation that “the complexity of image sequence is not a linear sum of the single-frame image complexity” [16]. This insight points toward a future where complexity metrics must account for temporal dynamics and multi-view information, a direction also highlighted in broader surveys of the field.

In summary, while significant progress has been made in developing descriptors for scene difficulty, the existing landscape is fragmented. Existing studies suggest that single metrics are often insufficient, and a unified, multi-dimensional, and detector-relevant scene complexity measure is still lacking. This gap directly motivates the development of the proposed Scene Detection Complexity (SDC) metric.

### 2.3. From Single-View Sensing to Multi-View and Multi-Modal Detection Systems

The need for scene-aware performance analysis becomes more evident as target perception systems evolve from single-view sensing to more complex multi-view and multi-frame settings. In the broader detection literature, single-camera systems have been studied for target detection and tracking in UAV platforms [18]. Multi-view and multi-camera methods have further been developed for robust camera orientation selection, feature projection, and 3D triangulation [19,20], as well as for occlusion-aware multi-camera multi-target detection [21].

In infrared target detection, the use of temporal information has also attracted increasing attention. Multi-frame track-before-detect strategies have shown clear advantages in weak-target tracking scenarios [22], while CNN-based multi-frame infrared small target detection methods further demonstrate the effectiveness of integrating spatial and temporal cues [23]. In addition, recent single-frame infrared small target detection methods based on high local variance, low-rank modeling, and sparse decomposition indicate that scene complexity remains a fundamental factor affecting target detectability even when more advanced detection models are employed [24]. These developments collectively suggest that detection systems are becoming increasingly sophisticated, which further motivates the need for a unified and interpretable metric for scene-induced detection difficulty.

### 2.4. Position of This Work

Unlike conventional single-indicator descriptors, the proposed scene detection complexity (SDC) metric is not intended to characterize only one isolated aspect of scene difficulty. Instead, it is designed to quantify scene-induced detection difficulty from a multi-dimensional perspective. In infrared small target detection, scene difficulty is jointly determined by target saliency, background complexity, and target–background coupling. A single indicator can usually reflect only one of these factors and may therefore become unreliable when different scene attributes interact. By integrating six complementary indicators with objective weighting, the proposed SDC provides a more comprehensive and balanced representation of scene difficulty. Therefore, the main advance of SDC over conventional feature-wise descriptors lies not merely in improved correlation values, but in its ability to establish a unified and interpretable metric for scene-level difficulty assessment across heterogeneous infrared sensing environments.

## 3. Materials and Methods

### 3.1. Basic Indicator Selection and Definition

The difficulty of infrared small target detection depends not only on the algorithm itself but also on the imaging scene. An effective scene-complexity metric should therefore describe the key factors that affect detectability from multiple perspectives. Based on previous studies and the physical characteristics of infrared images, six indicators are selected from three dimensions: target saliency, background complexity, and target–background coupling.

#### 3.1.1. Statistical Variance

Statistical variance reflects the global dispersion of image gray levels and can be used to describe the overall roughness of the background. A simple and homogeneous scene tends to yield a small SV value, whereas a cluttered scene usually results in a large value. It is defined as

(1) $$SV=\sqrt{\frac{1}{N}\sum_{i=1}^{N}{\left(x_{i}-\mu \right)}^{2}},$$

where *N* is the total number of pixels, $x_{i}$ is the gray value of the *i*th pixel, and μ is the mean gray level of the image.

#### 3.1.2. Target–Background Contrast

Target–background contrast directly reflects the gray-level saliency of the target with respect to its local background and is a key factor determining whether the target can be detected successfully. It is defined as

(2) $$TBC=\frac{\mu_{t}}{\mu_{b}},$$

where $\mu_{t}$ is the mean gray value of the target region and $\mu_{b}$ is the mean gray value of the local background region. The local background is defined as the area obtained by expanding the target bounding rectangle outward by twice its size.

#### 3.1.3. Signal-to-Clutter Ratio

The signal-to-clutter ratio is a commonly used indicator in infrared small target detection for measuring the contrast between the target and background clutter. It is defined as

(3) $$SCR=\frac{|\mu_{t}-\mu_{b}|}{\sigma_{b}},$$

where $\mu_{t}$ and $\mu_{b}$ denote the mean gray values of the target and background, respectively, and $\sigma_{b}$ is the standard deviation of the background gray values. Because a smaller SCR corresponds to greater detection difficulty, the transformed quantity ${SCR}^{\prime }=1-SCR_{\mathrm{norm}}$ is used in the final SDC construction.

#### 3.1.4. Information Entropy

Information entropy measures the richness and dispersion of gray-level information in an image. In infrared images, a larger entropy value usually indicates a more complex background and therefore a more difficult detection task. For an image of size M×N with *L* gray levels, entropy is defined as

(4) $$H=-\sum_{i=0}^{L-1}p_{i}\log_{2}p_{i},$$

where $p_{i}=n_{i}/\left(M\times N\right)$ and $n_{i}$ is the number of pixels at gray level *i*.

#### 3.1.5. Structural Similarity

Structural similarity is used to measure the structural resemblance between the target region and its local background. If the target and background exhibit high structural similarity, the target is more likely to be submerged in the background. Therefore, the transformed quantity ${SSIM}^{\prime }=1-SSIM$ is adopted as a positive indicator of detection difficulty. The original SSIM is computed as

(5) $$SSIM\left(T,B\right)=\frac{\left(2\mu_{t}\mu_{b}+C_{1}\right)\left(2\sigma_{tb}+C_{2}\right)}{\left(\mu_{t}^{2}+\mu_{b}^{2}+C_{1}\right)\left(\sigma_{t}^{2}+\sigma_{b}^{2}+C_{2}\right)},$$

where *T* and *B* denote the target and local background regions, $\mu_{t}$ and $\mu_{b}$ are the corresponding means, $\sigma_{t}$ and $\sigma_{b}$ are the standard deviations, $\sigma_{tb}$ is the covariance, and $C_{1}$ and $C_{2}$ are constants introduced to avoid zero denominators.

#### 3.1.6. Target Size

Target size is a direct factor affecting infrared small target detectability. Smaller targets contain fewer pixels and provide less usable intensity and structural information, which makes them more vulnerable to clutter interference [8]. Target size is defined as

(6) $$S=M_{t}\times N_{t},$$

where $M_{t}$ and $N_{t}$ are the height and width of the target bounding rectangle in pixels. For typical infrared small targets, *S* usually ranges from 1 to 81 pixels. Because larger targets are generally easier to detect, *S* is negatively correlated with detection difficulty and is directionally transformed during normalization.

### 3.2. Indicator Normalization

Because the six indicators have different physical units and numerical ranges, normalization is required before fusion. In this study, Min–Max normalization is adopted to map all indicators into the interval [0,1]. For indicators positively correlated with detection difficulty, such as SV and *H*, normalization is performed as

(7) $$x_{\mathrm{norm}}=\frac{x-x_{\min}}{x_{\max}-x_{\min}}.$$

For indicators negatively correlated with detection difficulty, such as TBC, SCR, SSIM, and *S*, a directional transformation is first applied and the transformed values are then normalized. The normalized indicators are denoted by ${SV}_{\mathrm{norm}}$, ${TBC}_{\mathrm{norm}}^{\prime }$, ${SCR}_{\mathrm{norm}}^{\prime }$, $H_{\mathrm{norm}}$, ${SSIM}_{\mathrm{norm}}^{\prime }$, and $S_{\mathrm{norm}}^{\prime }$.

### 3.3. Weight Estimation Based on the Entropy Weight Method

The entropy weight method is rooted in information theory. The basic idea is that an indicator with greater dispersion among samples contains more information and should therefore receive a higher weight. For an evaluation matrix $X={\left(x_{ij}\right)}_{m\times n}$ with *m* samples and *n* indicators, the proportion of the *i*th sample under the *j*th indicator is computed as $p_{ij}=x_{ij}/\sum_{i=1}^{m}x_{ij}$, and the entropy of the *j*th indicator is calculated as $e_{j}=-\left(1/\ln m\right)\sum_{i=1}^{m}p_{ij}\ln p_{ij}$. The corresponding entropy weight is

(8) $$w_{j}^{\left(E\right)}=\frac{1-e_{j}}{\sum_{j=1}^{n}\left(1-e_{j}\right)}.$$

### 3.4. Weight Estimation Based on Principal Component Analysis

PCA extracts the principal information of the original indicators through dimensionality reduction. The variance contribution ratio of each principal component reflects its information content, whereas the loadings of the original indicators reflect their contributions to the overall representation. For the standardized matrix $Z={\left(z_{ij}\right)}_{m\times n}$, the correlation matrix *R* is first calculated, followed by eigenvalue decomposition. The first *k* principal components are retained such that the cumulative variance contribution satisfies

(9) $$\frac{\sum_{p=1}^{k}\lambda_{p}}{\sum_{p=1}^{n}\lambda_{p}}\geq 85\%.$$

The composite score coefficient of the *j*th indicator is then defined as $c_{j}=\sum_{p=1}^{k}\theta_{p}\left|u_{jp}\right|$, where $\theta_{p}$ is the variance contribution ratio of the *p*th principal component and $u_{jp}$ is the corresponding loading. The normalized PCA weight is given by

(10) $$w_{j}^{\left(P\right)}=\frac{c_{j}}{\sum_{j=1}^{n}c_{j}}.$$

### 3.5. Comparison of the Two Weighting Methods

Although both the entropy weight method and PCA are objective weighting approaches, they follow different principles. The entropy weight method emphasizes indicator discrimination, whereas PCA emphasizes the internal correlation structure among indicators. Their main differences are summarized in Table 1.

In the present study, the six indicators are not independent. For example, TBC and SCR both describe differences between the target and its background, whereas entropy and SV both reflect aspects of background complexity. Therefore, using only one weighting method may lead to a biased representation. Combining the two methods allows both indicator discrimination and inter-indicator correlation to be taken into account.

### 3.6. Determination of the Composite Weight

To exploit the complementary strengths of the two methods, a linear combination is used to obtain the final composite weight:

(11) $$w_{j}=\alpha w_{j}^{\left(E\right)}+\left(1-\alpha \right)w_{j}^{\left(P\right)},\;j=1,2,\ldots ,n,$$

where α∈[0,1] is an adjustment coefficient. When α=1, only the entropy weight method is used; when α=0, only PCA is used.

Several strategies can be used to determine α, including equal weighting, expert consultation, and correlation-based selection. In this work, the correlation-based strategy is adopted. Specifically, the absolute Spearman correlation between the resulting SDC values and the mean detection performance of seven representative algorithms is evaluated for α ranging from 0 to 1 with a step size of 0.1. The maximum correlation is obtained at α=0.6 (0.883), which is therefore selected in this study.

### 3.7. Weighting Results

The MWIRSTD dataset contains 1053 images [25]. A subset of 200 images is selected for weight estimation, and the resulting weights are listed in Table 2.

The entropy term *H* has the largest weight (0.2524), indicating that background texture complexity is the most influential factor in scene difficulty. SCR′ and SSIM′ rank next, reflecting the importance of target–background energy contrast and structural coupling. By contrast, SV receives a relatively smaller weight, suggesting that global gray-level fluctuation alone is insufficient for comprehensive difficulty characterization.

### 3.8. Weight Stability Analysis

To assess the stability of the estimated weights, bootstrap resampling is conducted. A total of 200 samples are randomly drawn with replacement from the original set to form a new sample set, and the weighting process is repeated 100 times. The mean and standard deviation of each weight are reported in Table 3.

All standard deviations are below 0.012, indicating that the weighting results are stable with respect to sample perturbation. In particular, the entropy indicator shows the smallest standard deviation, which further supports its robustness as a core component of the proposed metric.

### 3.9. Definition of Scene Detection Complexity

Based on the six normalized indicators and the composite weights, the proposed SDC is defined as

(12) $$SDC=\sum_{j=1}^{6}w_{j}\;x_{j}^{\left(\mathrm{norm}\right)},$$

where $x_{j}^{\left(\mathrm{norm}\right)}$ denotes the normalized value of the *j*th indicator and $w_{j}$ is the corresponding composite weight, satisfying

(13) $$\sum_{j=1}^{6}w_{j}=1.$$

The SDC takes values in the interval [0,1], where a larger value indicates a more difficult detection scene.

**Figure 1:**
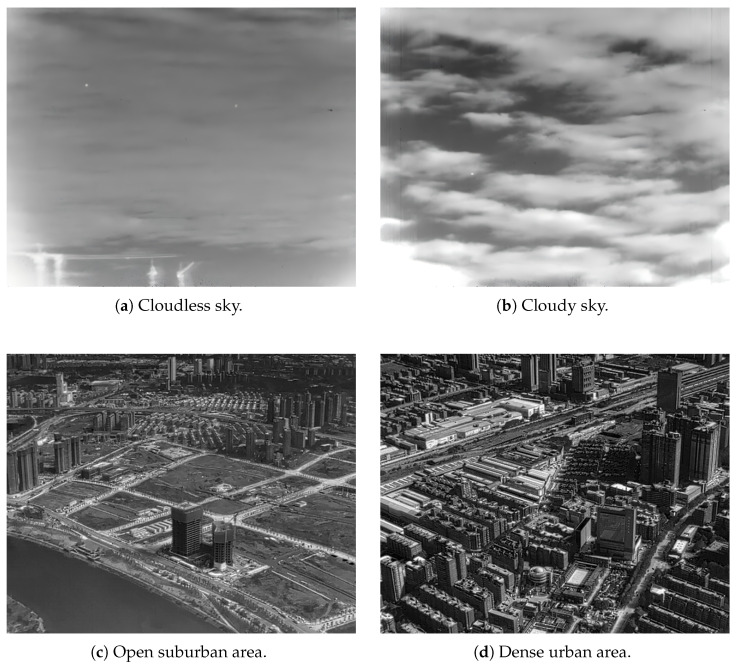
Representative infrared images with different scene detection complexities.

Representative examples are listed in Table 4, and four typical infrared images are shown in Figure 1. As the SDC increases, the scene becomes more complex and the target becomes more difficult to detect.

## 4. Experiments and Results

To verify the relationship between the proposed SDC metric and infrared small target detection performance, seven representative detection algorithms are evaluated on a unified test set. An image-level score (Score) is constructed by counting the number of algorithms that correctly detect the target on the same image. The correlations among SDC, subjective ratings, and objective detection performance are then analyzed.

### 4.1. Test Datasets

A total of 100 representative infrared images are selected from four public datasets. The six basic indicators and the corresponding SDC value are calculated for each image. All correlation analyses in this paper, including the correlations between SDC and subjective scores, between SDC and detection performance, and between SDC and individual indicators, are conducted on these 100 test images. The four images shown in Figure 1 and the examples listed in Table 5 are used only for qualitative illustration and do not replace the full statistical analysis. Although the 100-image test set covers multiple public datasets and diverse scene types, its overall size is still limited for a comprehensive assessment of generalization. Therefore, the present experimental results should be interpreted as a preliminary yet meaningful validation of the proposed SDC metric rather than a fully exhaustive benchmark. In particular, the current sample size may limit the statistical robustness of the conclusions under more diverse target categories, background structures, and imaging conditions. A larger-scale validation on more heterogeneous datasets will be conducted in future work to further examine the stability and generalizability of the proposed metric.

The datasets are summarized as follows.

- IRST640: infrared small target images with a resolution of 640×512, covering scenes such as sea surfaces, skies, and ground backgrounds [26].
- MSISTD: a multi-scene infrared small target dataset containing 1077 images with diverse scene conditions [27].
- SIRST-V2: a benchmark single-frame infrared small target dataset with a variety of complex backgrounds [28,29,30].
- Small-aircraft sequence dataset: an infrared weak small-aircraft detection and tracking dataset under ground and aerial backgrounds [31].

25 images are selected from each of the four public datasets to form the test datasets.

### 4.2. Compared Algorithms and Score Definition

To verify the applicability of SDC across different detection paradigms, seven representative infrared small target detection algorithms are selected, covering local contrast enhancement, morphological filtering, low-rank modeling, and deep learning. The compared methods are as follows: ADMD [32], LIG [33], Top-hat filtering, PSTNN [34], FKRW [35], DETR [36], and YOLO.

To evaluate the overall detectability of a single image across multiple detectors, an image-level score is defined. For the *i*th algorithm (i=1,2,…,7), if the target is correctly detected and no additional false alarms are produced, the binary outcome is set as $Score_{i}=1$; otherwise, $Score_{i}=0$. A detection is regarded as correct when the predicted target location falls within the annotated target neighborhood and no extra false alarm is produced in the image. The overall score of an image is then given by

(14) $$Score=\sum_{i=1}^{7}Score_{i}.$$

Therefore, Score indicates how many of the seven representative algorithms can correctly detect the target in a given scene. A larger Score implies that the scene is easier for detection, whereas a smaller Score indicates higher difficulty. Although this score does not describe fine-grained performance differences among algorithms, it provides an intuitive image-level measure that is well suited to the correlation analysis in this study.

### 4.3. Qualitative Examples

To illustrate the relationship between SDC and detection difficulty, four representative images are selected from the full set of 100 test images. Table 5 lists the corresponding SDC values, subjective difficulty ratings (averaged from five researchers in infrared image processing), and the detection outcomes of the seven algorithms.

The qualitative examples reveal a consistent trend across algorithms. Traditional methods such as ADMD, LIG, Top-hat, and FKRW generally perform well in low-complexity scenes but degrade noticeably as the SDC increases, indicating a stronger sensitivity to background clutter. By contrast, deep-learning-based methods, such as DETR and YOLO, maintain a certain level of detection capability even in highly complex scenes, which suggests better robustness and generalization. PSTNN exhibits comparatively stable behavior across different scene complexities, indicating the effectiveness of low-rank decomposition in suppressing clutter.

### 4.4. Correlation Analysis Between SDC and Detection Performance

To quantitatively evaluate the relationship between SDC and detection performance, four statistical measures recommended by the Video Quality Experts Group are adopted: the Pearson linear correlation coefficient (PLCC), the Spearman rank-order correlation coefficient (SROCC), the Kendall rank-order correlation coefficient (KROCC), and the root mean square error (RMSE). PLCC measures linear correlation, SROCC and KROCC describe monotonic correlation, and RMSE reflects prediction error.

#### 4.4.1. Correlation Between SDC and Subjective Ratings

The first analysis evaluates whether the proposed SDC is consistent with human perception of detection difficulty. Based on the full set of 100 test images, the correlations between SDC and the mean subjective scores assigned by five researchers are

PLCC=0.956,SROCC=0.943,KROCC=0.821,RMSE=0.314.

These results indicate a strong positive relationship between SDC and subjective difficulty perception, which suggests that the proposed metric aligns well with human judgment.

#### 4.4.2. Correlation Between SDC and Algorithmic Detection Performance

The relationship between SDC and the image-level Score is then evaluated on the same 100-image test set. The resulting statistics are

PLCC=−0.902,SROCC=−0.917,KROCC=−0.786,RMSE=0.873.

**Figure 2:**
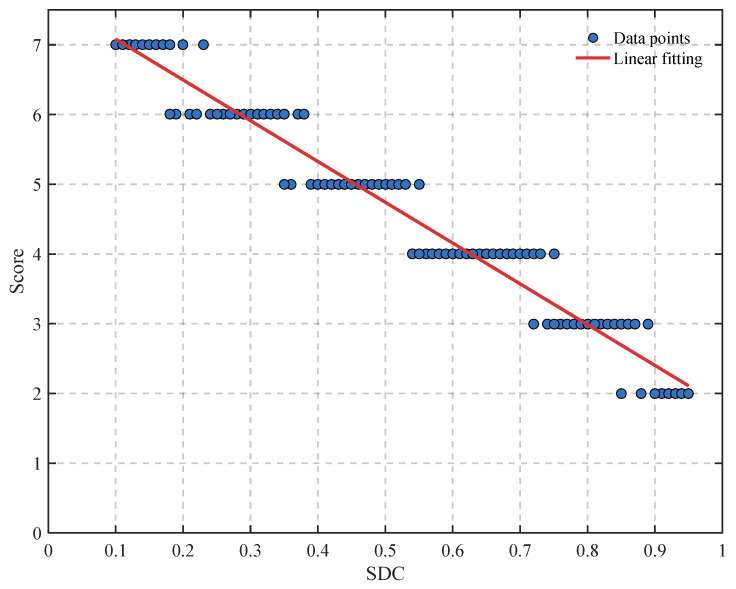
Relationship between SDC and Score on the test datasets.

The large negative PLCC and SROCC values show that higher SDC values correspond to fewer algorithms successfully detecting the target. In other words, scenes with larger SDC values are indeed more difficult for detection. The negative relationship between SDC and Score over the whole 100 test images is illustrated in Figure 2.

#### 4.4.3. Comparison Between SDC and Individual Indicators

To demonstrate the necessity of multi-index fusion and the superiority of SDC, we conducted correlation analyses between SDC and each individual index, as well as between SDC and Score. The results are shown in the table below. Specifically, for TBC, SCR, SSIM, and S, the correlation coefficients listed in Table 6 represent the original indicators (negatively correlated with difficulty); for the other indicators, they are the complements that are positively correlated with difficulty.

It shows that: 1. The absolute PLCC and SROCC values of SDC are larger than those of any single indicator, which demonstrates that the proposed composite metric captures detection difficulty more comprehensively than any individual feature.

2. Although SV, *H*, and SSIM are negatively correlated with Score, their absolute correlations remain below 0.70.

3. The original values of TBC and SCR are positively correlated with Score, which is in line with intuition (the higher the contrast and the higher the signal-to-noise ratio, the easier it is to detect), but when used alone, the correlation is also lower than that of SDC.

## 5. Discussion

The above experimental results indicate that the scene detection complexity (SDC) proposed in this paper can effectively quantify the difficulty of detecting weak and small targets in infrared images. The main advantages are as follows:

1. Highly consistent with subjective perception: The PLCC between SDC and expert subjective ratings reached 0.956, indicating that it aligns with human perception of the complexity of the scene.

2. Highly correlated with objective detection performance: The PLCC between SDC and Score reached −0.902, indicating a strong negative correlation. This verifies the core assumption that “the higher the complexity, the more difficult the detection.”

3. Superiority over a single indicator: The correlation of SDC is significantly higher than that of any single indicator, demonstrating the necessity of multi-dimensional integration.

From the perspective of algorithm types, the traditional methods (ADMD, LIG, Top-hat, FKRW) are highly sensitive to the SDC value and their performance significantly deteriorates when SDC is greater than 0.6; while the deep learning methods (DETR, YOLO) can still maintain certain detection capabilities when SDC is greater than 0.7, demonstrating better generalization ability. This finding can provide a reference for algorithm selection in practical engineering: in simple scenarios (SDC < 0.4), traditional methods with lower computational complexity can be chosen; in complex scenarios (SDC > 0.6), deep learning methods should be given priority.

Nevertheless, these observations are currently derived from a 100-image test set and should be interpreted with appropriate caution. Although the selected samples cover multiple datasets, further validation on larger and more diverse data collections is still necessary to confirm the robustness of the observed trends.

## 6. Conclusions

This paper proposed a scene detection complexity metric, termed SDC, to address the lack of a unified quantitative description of scene difficulty in infrared small target detection. The metric integrates six indicators from three aspects—target saliency, background complexity, and target–background coupling—and combines the entropy weight method with PCA to obtain objective composite weights. The resulting scalar complexity index takes values in [0,1] and provides an interpretable measure of scene difficulty.

The main significance of this work lies in providing a scene-centered analytical tool for infrared small target detection. The proposed SDC can be used to pre-assess scene difficulty before detector deployment, support algorithm selection, analyze performance boundaries, and improve the interpretability of benchmark evaluations. Rather than merely describing algorithm outputs, it helps answer a more general question: what kinds of scenes are intrinsically challenging for infrared small target detection?

This study still has several limitations. First, although the proposed SDC metric is validated on 100 representative images selected from multiple public datasets, the overall test set remains limited in size and diversity. As a result, the current conclusions should be regarded as a preliminary validation rather than a fully exhaustive assessment of generalization across all infrared small target scenarios. Second, the image-level Score defined in this paper is intended to reflect the overall detectability of a scene across multiple representative algorithms, but it does not capture more fine-grained differences in detector behavior, such as precision–recall trade-offs or sensitivity to different false-alarm patterns. Third, the current SDC formulation is mainly established for single-target, single-frame infrared images and does not explicitly incorporate temporal motion cues, multi-target interactions, or cross-sensor imaging variations. These aspects may also influence scene-induced detection difficulty and deserve further investigation.

Future work will therefore focus on three directions. First, the proposed SDC will be validated on larger and more diverse datasets involving richer target categories, scene types, and imaging conditions, so as to further assess its statistical robustness and generalization capability. Second, more detailed evaluation measures, such as precision, recall, F1-score, and AUC, will be incorporated to establish a finer mapping between scene complexity and actual detector performance. Third, the proposed metric will be extended to more complex infrared sensing tasks, including multi-target detection, target tracking, simulated image quality evaluation, and data selection and augmentation.

## Figures and Tables

**Table 1:** Comparison between the entropy weight method and PCA.

Criterion	Entropy Weight Method	Principal Component Analysis
Theoretical basis	Information theory (entropy)	Multivariate statistical analysis (variance decomposition)
Weighting basis	Indicator dispersion (discriminability)	Correlation structure among indicators (representativeness)
Data requirement	Indicator values only	Correlation analysis among indicators
Advantage	Highlights discrimination and is easy to implement	Reduces information redundancy and reflects structural relationships
Limitation	Ignores correlation and may introduce repeated weighting	Sensitive to outliers and requires sufficient sample size
Applicable scenario	Weak correlation among indicators	Strong correlation among indicators

**Table 2:** Weighting results for the six indicators.

Method	SV	TBC′	SCR′	*H*	SSIM′	*S*′
Entropy weight $w_{j}^{\left(E\right)}$	0.1523	0.1846	0.2135	0.2617	0.1879	0.1055
PCA weight $w_{j}^{\left(P\right)}$	0.1368	0.2072	0.1954	0.2385	0.2221	0.1174
Composite weight $w_{j}$ (α=0.6)	0.1461	0.1936	0.2063	0.2524	0.2016	0.1083

**Table 3:** Weight stability analysis using bootstrap resampling.

Statistic	SV	TBC′	SCR′	*H*	SSIM′	*S*′
Mean	0.1458	0.1942	0.2059	0.2521	0.2020	0.1079
Standard deviation	0.0087	0.0102	0.0095	0.0076	0.0113	0.0065

**Table 4:** Examples of SDC values for representative infrared scenes.

Scene Type	SDC	Description
Cloudless sky	0.12	Uniform background, salient target, very easy to detect
Cloudy sky	0.38	Limited clutter, target still relatively salient
Open suburban area	0.64	Strong clutter, target partially submerged
Dense urban area	0.91	Highly cluttered background, target almost invisible

**Table 5:** Detection results and SDC values for four representative test images.

Image	SDC	Subjective Score	ADMD	LIG	Top-Hat	PSTNN	FKRW	DETR	YOLO	Score
a	0.12	1	✓	✓	✓	✓	✓	✓	✓	7
b	0.38	1.5	✓	✓	×	✓	✓	✓	✓	6
c	0.64	2.5	×	✓	×	✓	✓	✓	✓	5
d	0.91	3.5	✓	×	×	✓	×	✓	✓	4

Note: ✓ indicates that the corresponding algorithm correctly detects the target in the image without extra false alarms, whereas × indicates that it fails to do so.

**Table 6:** Correlation coefficients between different indicators and the image-level Score.

Indicator	SV	TBC	SCR	*H*	SSIM	*S*′	SDC
PLCC	−0.631	+0.724 *	+0.753 *	−0.695	−0.668	−0.580	−0.902
SROCC	−0.618	+0.709 *	+0.741 *	−0.682	−0.654	−0.560	−0.917

* The original values of TBC and SCR are positively correlated with Score because higher contrast and higher signal-to-clutter ratio generally make targets easier to detect. In the construction of SDC, these indicators are directionally transformed to maintain consistency with the definition of detection difficulty.

## Data Availability

The raw data supporting the conclusions of this article will be made available by the authors on request.
